# Changes in cardio-respiratory-vascular coupling dynamics across altitude gradients: a shift from linear synchrony to nonlinear complexity

**DOI:** 10.3389/fphys.2026.1818880

**Published:** 2026-04-23

**Authors:** Hongyun Liu, Shijing Wu, Ping Zhan, Xiaohua Yu, Yiming Kuang, Yi Han, Hongyue Zu, Guojing Wang, Weidong Wang

**Affiliations:** 1 Medical Innovation Research Division, Chinese People's Liberation Army (PLA), General Hospital, Beijing, China; 2 Key Laboratory of Biomedical Engineering and Translational Medicine, Ministry of Industry and Information Technology, Beijing, China

**Keywords:** altitude gradient, autonomic regulation, cardio-respiratory-vascular coupling, hypoxic stress, long-term high-altitude acclimatization

## Abstract

**Introduction:**

Prolonged high-altitude hypoxia induces acclimatization-related changes of multiple human physiological systems. The cardio-respiratory-vascular (CRV) coupling system, a core homeostasis-maintaining integrative mechanism, has incompletely elucidated altitude-acclimatization-related patterns. This study aimed to systematically explore CRV coupling and related physiological parameter changes across a broad altitude range in long-term acclimatized individuals.

**Methods:**

A multicenter cross-sectional study was conducted across five altitude gradients (HA0: <100 m, n=62; HA1: 1300 m, n=74; HA2: 3700 m, n=60; HA3: 4300 m, n=71; HA4: 5100 m, n=74). Synchronous electrocardiogram, hemodynamic, and respiratory signals were collected from healthy adults with ≥3 months of acclimatization. CRV coupling strength and complexity were computed, integrated with heart rate variability (HRV), hemodynamic, and respiratory parameters for analysis, with a HA4-specific physiological correlation network constructed.

**Results:**

CRV coupling strength showed altitude-dependent attenuation, while complexity increased with elevation. At HA4, HRV indices changed distinctly: Mean RR, logHF, and SampEn decreased (P<0.0001), and LF/HF increased (P<0.0001). SpO_2_ declined and PPV rose with altitude; DBP (P = 0.0042) and MAP (P = 0.0204) increased only at HA4. Respiratory parameters exhibited asymmetric characteristics: AED and BR elevated (P<0.05), with BR_CV and EDC_CV increasing significantly only at HA4 (P<0.01). At HA4, coupling strength correlated positively with vagal activity/heart rate complexity and negatively with sympathetic dominance/respiratory variability, while complexity correlated positively with sympathetic dominance.

**Discussion:**

Long-term high-altitude acclimatization is characterized by a regulatory shift of the CRV system from strength-dominant linear synchrony to complexity-prioritized nonlinear flexibility. Extreme hypoxia may trigger enhanced nonlinear interactions to compensate for reduced linear synchrony, relying on respiratory rhythm stabilization and precise autonomic balance modulation. The findings may provide novel insights into the integrated physiology of high-altitude acclimatization.

## 1 Introduction

Life at high altitude demands continuous physiological negotiation with hypobaric hypoxia. Long-term residents undergo acclimatization-related changes across cardiovascular, respiratory, and autonomic systems that is essential for preserving oxygen homeostasis (Nuss, 2022; Richalet et al., 2024; Prabhakar and Semenza, 2015; Prosperi et al., 2023; Mohanty and Ahmad, 2025). Crucially, these systems do not function independently. Instead, they operate as a dynamically coupled network, coordinating precisely to optimize oxygen uptake, transport, and utilization (Kitney et al., 1985; Censi et al., 2003; Tikhonova et al., 2022; Crystal and Pagel, 2020). Cardio-respiratory-vascular (CRV) coupling is a core physiological mechanism for maintaining oxygen homeostasis. CRV dynamic characteristics are closely related to autonomic regulation, respiratory rhythm stability (Kaminsky et al., 2023), and cardiac dynamics (Suth et al., 2024; Tse et al., 2016). Abnormal changes in coupling metrics have been confirmed in pathological states (Lin et al., 2020; Ma et al., 2024). Many studies have documented altitude-related changes in isolated physiological metrics. These include heart rate variability (HRV) (Hou et al., 2023; Cornolo et al., 2004), breathing patterns (Burtscher et al., 2025; Furian et al., 2022; Wang et al., 2024), and blood pressure regulation (Zhang et al., 2022; Narvaez-Guerra et al., 2018; Gatterer et al., 2024). However, our integrated understanding of how the CRV systems interact as a coherent network remains fragmented. In particular, how this network evolves across altitude gradients in long-term acclimatized populations, and through what regulatory mechanisms, is still poorly mapped.

Progress in the field has been constrained by two predominant limitations. First, research has predominantly focused on acute physiological stress responses to high-altitude exposure (hours to weeks) (Parati et al., 2018; Bärtsch and Swenson, 2013; Luks et al., 2017; Luks et al., 2024), leaving both the established steady state of chronically adapted individuals and the systemic logic that sustains it far less clear. Second, conventional approaches often analyze output variables from single systems (Hou et al., 2023; Cornolo et al., 2004; Burtscher et al., 2025; Furian et al., 2022; Zhang et al., 2022; Narvaez-Guerra et al., 2018; Gatterer et al., 2024), failing to capture the dynamics of cross-system interaction. At present, classic methods for evaluating cardiorespiratory coupling include respiratory sinus arrhythmia (RSA) (Menuet et al., 2025), phase synchronization analysis (Bartsch et al., 2012), time-delay stability analysis (Bashan et al., 2012), and cardiorespiratory coordination analysis (Garcia-Retortillo et al., 2019). While these methods have provided valuable insights, they often focus on pairwise interactions rather than the integrated coupling among multiple systems. The present study adopts a multimodal coupling analysis (MMCA) framework that simultaneously captures interactions among systems, allowing for a more comprehensive assessment of network-level dynamics (Lin et al., 2020). The MMCA can quantify both coupling strength (reflecting linear synchrony) and complexity (characterizing flexible, nonlinear interplay) simultaneously, which is more suitable for comprehensively revealing the overall coupling dynamics of the CRV system under high altitude. Furthermore, this framework has delivered key insights into chronic conditions like aging and heart failure (Lin et al., 2020; Ma et al., 2024), yet its application to high-altitude physiology, specifically for probing CRV coupling in long-term residents, remains notably scarce.

The coupling dynamics of the CRV system are the core components of the human physiological network. Network physiology focuses on the dynamic coordination laws between various physiological systems and subsystems. The coordination characteristics changes in different physiological states (Ivanov, 2021; Bashan et al., 2012). The dynamic coupling of physiological networks is closely related to the body’s adaptation to external environmental stress, and its characteristic changes can reflect the physiological adjustment state of the body under hypoxic stress. The evidence suggest that acclimatization from lowlands to very high altitude does not follow a simple linear trajectory but is characterized by critical thresholds and strategic regulatory changes (Storz and Scott, 2019; Storz, 2021; Ortiz-Prado et al., 2024). It remains to be determined how coupling within the CRV network changes across this continuum. At one end, coupling may shift toward tighter synergy, which enhances metabolic efficiency. At the other end, it may shift toward decoupling and instability, which increases physiological strain. Furthermore, the links between these coupling dynamics and concurrent shifts in autonomic balance, hemodynamic stability, and respiratory control require systematic clarification. Addressing these questions is central to elucidating the integrated physiology of high-altitude acclimatization and to identifying markers of decompensation risk.

We therefore conducted a cross-sectional study across five altitude gradients. These ranged from lowland (<100 m) to 5100 m. At each altitude level, we enrolled healthy long-term residents. Through analysis of synchronous heart rate, respiratory and blood pressure signals, we quantified altitude-related changes in coupling strength and complexity across the CRV network. Combining these metrics with comprehensive HRV, hemodynamic, and respiratory profiles allowed us to model the acclimatization-related regulatory landscape. The work aimed to describe altitudinal characteristics in CRV coupling dynamics, to determine their relationship with autonomic, hemodynamic, and respiratory control, and to identify features of acclimatization-related network changes or even risk signatures under high altitude stress. Moving beyond single-system analysis, this work provides an integrative, dynamic perspective on long-term high altitude acclimatization, with implications for protecting health and predicting clinical risk in highland populations (Leacy, et al., 2021; Wang et al., 2025).

## 2 Material and methods

### 2.1 Study design and participants

This investigation employed a multi-altitude cross-sectional design. Participant enrollment and data collection were conducted across five distinct altitude gradient: a lowland plain site (HA0, <100 m), and four high altitude sites at approximately 1300 m (HA1), 3700 m (HA2), 4300 m (HA3), and 5100 m (HA4).

At each site, we recruited unrelated, healthy Han Chinese male volunteers following a standardized health screening. The subjects in this study are healthy people who have lived at the corresponding altitude for ≥3 months, which meets the basic threshold of hypoxic acclimatization. Their physiological characteristics reflect the CRV coupling changes under hypoxic acclimatization, rather than the adaptive changes at the long-term evolutionary level. The primary inclusion criteria were: (1) continuous residency at the respective altitude for a minimum of three months prior to the study, with no significant altitude change (>300 m) in the preceding two weeks; (2) age between 18 and 40 years; (3) no known history of chronic cardiovascular, respiratory, or neurological diseases; (4) normal blood pressure (systolic < 140 mmHg and diastolic < 90 mmHg); and (5) light smoking habits (<5 cigarettes/day) and no history of alcohol abuse.

Female participants were excluded to avoid potential confounding effects of menstrual cycle and hormonal fluctuations on autonomic nervous activity and CRV coupling dynamics, ensuring physiological homogeneity within each altitude group. Potential participants were excluded if they met any of the following conditions: (1) presence of acute illnesses such as infection or fever at the time of assessment; (2) any documented history of cardiopulmonary or neurological disorders; (3) current use of medications known to affect cardiovascular or autonomic nervous system function; (4) diagnosed psychiatric or cognitive conditions; or (5) recent heavy consumption of tobacco, alcohol, tea, or coffee, or chronic use of any medication.

The study protocol received ethical approval from the Ethics Committee of the Chinese PLA General Hospital (Approval No.: S2025-885-01) and was registered with the Chinese Clinical Trial Registry (Registration No.: ChiCTR2500112985). All volunteers provided written informed consent prior to their participation.

### 2.2 Data acquisition

All measurements followed a unified standardized operating procedure across all centers to ensure data consistency. Experiment were conducted between 09:00 and 11:00 to minimize potential confounding effects of circadian rhythms. The laboratory conditions were maintained at a comfortable ambient temperature (22-25) °C and relative humidity (20-70)% across all study sites. Participants were positioned supine on a medical bed and asked to remain still, keep their eyes closed, and maintain quiet, natural breathing. Following a 5-minute adaptation period for stabilization, a 5-minute recording of synchronous multi-modal physiological signals was subsequently acquired for analysis. This 5-minute duration conforms to international guidelines for HRV analysis (Task Force of the European Society of Cardiology the North American Society of Pacing, 1996), sliding window processing was applied to improve the stability of nonlinear dynamic indices.

Synchronous data acquisition was performed using an integrated BIOPAC MP160 system (Biopac Systems Inc., Goleta, CA, USA) and a CNAP Monitor 500 (CNSystems Medizintechnik, Graz, Austria). The recorded signals included single-lead electrocardiogram (ECG), respiration (RSP), continuous hemodynamic waveforms [continuous blood pressure (CBP), mean arterial pressure (MAP), cardiac output (CO), pulse pressure variation (PPV)], and peripheral oxygen saturation (SpO_2_).

For ECG, a Lead II configuration consistent with standard 12-lead Holter monitoring was used. The negative electrode was placed below the midpoint of the right clavicle, the positive electrode was placed below the left costal margin, and the ground electrode was placed below the right costal margin. RSP was captured via a chest-mounted transducer that recorded thoracic expansion and contraction. Continuous non-invasive hemodynamic monitoring was achieved using a finger cuff sensor placed on the index and middle fingers of the left hand, complemented by a standard upper-arm cuff positioned 2–3 cm above the antecubital fossa on the same arm. All physiological signals were sampled at 2000 Hz. The raw data were stored in MAT-file format for subsequent offline processing and analysis.

### 2.3 Signal preprocessing

The preprocessing of multi-modal physiological signals was carried out in MATLAB R2020 (MathWorks, Natick, MA, USA) using a combination of built-in functions and custom-developed scripts. As a first step, a 50-Hz notch filter was applied uniformly to all recorded signals to eliminate power-line interference.

The ECG signal was band-pass filtered (0.5-40)Hz. QRS complexes were then identified, and their peaks were detected using Kubios HRV software (version 3.4, Kubios Oy, Kuopio, Finland). From these fiducial points, a raw series of R-R intervals (RRI) was constructed. This series underwent manual inspection and cleaning. Ectopic beats, defined as intervals exceeding 2000 ms, falling below 300 ms, showing an abrupt change >200 ms from the preceding interval, or deviating by more than 20% from the local mean of the five prior intervals, were excluded (Liu et al., 2020). The resulting, cleaned RRI series was finally resampled at 8 Hz via cubic spline interpolation to generate a uniformly sampled HRV time series. This 8 Hz resampling follows the original MMCA methodology (Lin et al., 2020), which employs cubic interpolation at 0.125 s intervals to satisfy the Nyquist theorem while preserving waveform information for subsequent phase and amplitude analyzes. The same resampling frequency was also applied to respiratory and blood pressure signals to ensure temporal alignment across modalities.

The raw RSP waveform was first resampled to 8 Hz. To isolate the component related to breathing effort, a first-order Butterworth band-pass filter with cutoff frequencies of 0.01 and 2 Hz was applied (Romano et al., 2023). The derivative of the filtered signal was subsequently computed and normalized to the range (−1, 1). This transformation yields positive values during the inspiratory phase and negative values during expiration.

The raw CBP waveform was conditioned through a band-pass filter (0.5-12)Hz to remove baseline drift and high-frequency artifacts. Signal quality was further enhanced by applying a 50-ms moving average filter with a 9-Hz cutoff to suppress residual high-frequency noise. Beat-by-beat detection of waveform features, including the onset (diastolic blood pressure, DBP) and peak (systolic blood pressure, SBP) of each pulse, was performed using a custom MATLAB function, followed by visual verification (Li et al., 2010). The extracted SBP and DBP time series were then resampled to 8 Hz to match the sampling rate of the processed HRV and RSP signals for subsequent synchronous analysis.

### 2.4 Feature extraction

#### 2.4.1 Quantification of CRV coupling

The coupling dynamics among RSP, RRI, SBP, and DBP were quantified using the multimodal coupling analysis (MMCA) framework (Lin et al., 2020). The procedure consisted of the following four steps.

##### 2.4.1.1 Step 1:extraction of intrinsic mode functions

The time series for RSP, RRI, SBP, and DBP were each decomposed via the ensemble empirical mode decomposition (EEMD) method. Gaussian white noise with an amplitude of 0.2 times the standard deviation of the signal was added, and the ensemble was set to 100 iterations (Equations 1, 2). This process yielded a set of IMFs for each signal.

(1) $${\text{s}}_{\text{i}}(\text{t})=\text{s}(\text{t})+{\text{w}}_{\text{i}}(\text{t})$$

(2) $${\text{s}}_{\text{i}}(\text{t})=\sum_{\text{j}=1}^{\text{n}}{\text{c}}_{\text{j}}(\text{t})+{\text{r}}_{\text{n}}(\text{t})$$

s(t) and s_i_(t) represent the original and noise-augmented time series, respectively. c_j_(t) denotes the *j-th* IMF, and r_n_(t) is the residual after extracting *n-th* IMF.

##### 2.4.1.2 Step 2: identification of dominant and frequency-matched IMFs

For the RSP signal, the IMF with the highest energy was selected as the dominant RSP-IMF, which captures the primary oscillatory component while mitigating non-stationary interference (Lin et al., 2020). The Hilbert transform (Equations 3, 4) was then applied to all IMFs from the RRI, SBP, and DBP series, as well as to the dominant RSP-IMF, to compute their instantaneous amplitude A(t), and phase φ(t). P denotes the Cauchy principal value.

(3) $$\tilde{\text{x}}(\text{t})=\frac{1}{\text{π}}\text{P}\int \frac{\text{x}(\text{τ})}{\text{t}-\text{τ}}\text{dτ}$$

(4) $$\text{z}(\text{t})=\text{x}(\text{t})+\text{i}\cdot \tilde{\text{x}}(\text{t})=\text{A}(\text{t}){\text{e}}^{\text{jφ}(\text{t})}$$

(5) $$\text{f}(\text{t})=\frac{\text{d}}{\text{dt}}\text{φ}(\text{t})$$

Subsequently, for RRI, SBP, and DBP, the specific IMF whose mean instantaneous frequency (Equation 5) was closest to that of the dominant RSP-IMF was identified as the frequency-matched IMF. These matched components are considered to represent the specific modulations of heart rate and blood pressure by respiration, isolating them from other physiological influences.

##### 2.4.1.3 Step 3: computation of phase synchronization index

A sliding window of length T = 50 seconds was applied to segment the IMFs. Within each window *i* ( ${\text{t}}_{\text{i}}-\frac{\text{T}}{2}\leq \text{t}\leq {\text{t}}_{\text{i}}+\frac{\text{T}}{2}$), the phase synchronization index ${\text{ρ}}_{\text{i}}$ between pairwise combinations of the dominant RSP-IMF and the frequency-matched RRI, SBP, and DBP IMFs was calculated using Equation 6. The window was stepped in 1-second increments across the entire signal, generating time-varying synchronization series ρ_RSP-RRI_(t_i_), ρ_RSP-SBP_(t_i_),ρ_RSP-DBP_(t_i_), ρ_SBP-RRI_(t_i_), and ρ_DBP-RRI_(t_i_).

(6) $${\text{ρ}}_{\text{i}}=\frac{1}{\text{T}}|\int_{{\text{t}}_{\text{i}}-\frac{\text{T}}{2}}^{{\text{t}}_{\text{i}}+\frac{\text{T}}{2}}{\text{e}}^{\text{jΔφ}(\text{t})}\text{dt}|$$

Δφ(t) represents the instantaneous phase difference between two IMFs. The index ${\text{ρ}}_{\text{i}}$ ranges from 0 to 1, with higher values indicating stronger synchrony (Lin et al., 2020; Ma et al., 2024).

##### 2.4.1.4 Step 4: quantification of coupling metrics

The mean of each phase synchronization time series was calculated, yielding five average coupling strength metrics including ρ_RSP-RRI_, ρ_RSP-SBP_, ρ_RSP-DBP_, ρ_SBP-RRI_, and ρ_DBP-RRI_. To characterize the complexity of these interactions, the refined composite multiscale entropy (RCMSE) (Hermann, 2024) was computed for each synchronization time series, resulting in corresponding complexity metrics C_RSP-RRI_, C_RSP-SBP_, C_RSP-DBP_, C_SBP-RRI_, and C_DBP-RRI_. Finally, overall indices for total CRV coupling strength ${\text{ρ}}_{\text{total}}$) and complexity ( ${\text{C}}_{\text{total}}$) were derived by integrating the pairwise metrics according to Equations 7 and 8.

(7) $${\text{ρ}}_{\text{total}}={\text{ρ}}_{\text{RSP}-\text{RRI}}+\frac{{\text{ρ}}_{\text{RSP}-\text{SBP}}+{\text{ρ}}_{\text{RSP}-\text{DBP}}}{2}+\;\frac{{\text{ρ}}_{\text{SBP}-\text{RRI}}+{\text{ρ}}_{\text{DBP}-\text{RRI}}}{2}$$

(8) $${\text{C}}_{\text{total}}={\text{C}}_{\text{RSP}-\text{RRI}}+\frac{{\text{C}}_{\text{RSP}-\text{SBP}}+{\text{C}}_{\text{RSP}-\text{DBP}}}{2}+\frac{{\text{C}}_{\text{SBP}-\text{RRI}}+{\text{C}}_{\text{DBP}-\text{RRI}}}{2}$$

#### 2.4.2 HRV analysis

Time-domain HRV indices were computed according to international guidelines (Task Force of the European Society of Cardiology the North American Society of Pacing, 1996), including mean RRI (Mean RR), and the standard deviation of NN intervals (SDNN). For frequency-domain analysis, the RRI series was resampled at 4 Hz. The 4 Hz resampling is a standard requirement for HRV frequency-domain analysis to avoid spectral aliasing, while the earlier 8 Hz resampling supports high-precision coupling dynamic analysis. Power spectral density was estimated via fast Fourier transform to quantify low-frequency power (LF, 0.04-0.15 Hz), high-frequency power (HF, 0.15-0.40 Hz), and the LF/HF ratio. Natural logarithms (log) of the LF and HF power values were used because of the skewness of their distributions. This study uses the LF/HF ratio as an index reflecting relative autonomic influences. It should be noted that this index cannot completely and directly reflect the individual activity of sympathetic and vagal nerves, and its result interpretation needs to be comprehensively analyzed in combination with other HRV indexes (Cornolo et al., 2004; Hou et al., 2023). Additionally, sample entropy (SampEn) was calculated as a nonlinear metric to assess the irregularity and complexity of the HRV time series (Castiglioni et al., 2023).

#### 2.4.3 Hemodynamic analysis

Beat-to-beat time series of SBP and DBP, derived from the preprocessed CBP waveform, were analyzed alongside continuous recordings of MAP, CO, PPV, and SpO_2_. The mean and standard error of these parameters were calculated as the primary hemodynamic features.

#### 2.4.4 RSP analysis

Verification confirmed the subjects’ breathing rates and ensured that the fixed HF band adequately captured respiration-related vagal modulation. The preprocessed RSP was analyzed using the open-source BreathMetrics MATLAB toolbox (https://github.com/zelanolab/breathmetrics). Extracted respiratory features including average exhale duration (AED), average inhale duration (AID), breathing rate (BR), coefficient of variation for breathing rate (BR_CV), coefficient of variation for exhale duty cycle (EDC_CV), and coefficient of variation for inhale duty cycle (IDC_CV).

### 2.5 Statistical analysis

Data analysis and graphical presentation were performed using SPSS version 26.0 (SPSS Inc., Chicago, IL, USA) and GraphPad Prism version 10.0 (GraphPad Software, Boston, MA, USA). Continuous variables are expressed as mean ± standard error. Differences among altitude groups were assessed using one-way analysis of variance (ANOVA), with *post-hoc* multiple comparisons corrected by the Bonferroni method to control for type I error. Spearman’s rank correlation analysis was employed to explore relationships between multi-system coupling indices and HRV, hemodynamic, and respiratory parameters at HA4. Effect sizes were reflected by mean differences and variance analysis. A two-sided p-value <0.05 was considered statistically significant.

## 3 Results

### 3.1 Characteristics of the study population

The study enrolled a total of 341 healthy adults, distributed across the five altitude gradients as follows: HA0 (n=62), HA1 (n=74), HA2 (n=60), HA3 (n=71), and HA4 (n=74). All participants completed the testing protocol without experiencing severe altitude-related complications, such as pulmonary or cerebral edema, and no tests were terminated prematurely.

Demographic characteristics for the five groups are summarized in **Table 1**. One-way ANOVA revealed a statistically significant difference in age among the groups (F = 5.123, P = 0.0005). *Post-hoc* pairwise comparisons with Bonferroni correction indicated that participants in the HA2 group were significantly older than those in HA1 (P = 0.0002), and a similar age difference was observed between the HA4 and HA1 groups (P = 0.0168). In contrast, no significant inter-group differences were found for height (F = 1.745, P>0.05), weight (F = 1.871, P>0.05), or body mass index (BMI; F = 1.525, P>0.05).

**Table 1:** Demographic characteristics of subjects in five altitude groups.

Variables	HA0 (n=62)	HA1 (n=74)	HA2 (n=60)	HA3 (n=71)	HA4 (n=74)	F	P
Age, year	21.4 ± 0.4	20.6 ± 0.2	22.2 ± 0.3b***	21.6 ± 0.3	21.6 ± 0.4b*	5.123	0.0005
Height, cm	175.6 ± 0.7	174.8 ± 0.8	173.3 ± 0.7	174.8 ± 0.6	173.4 ± 0.7	1.745	0.1396
Weight, kg	73.4 ± 1.0	70.1 ± 1.1	67.9 ± 1.4	65.2 ± 1.1	70.4 ± 1.0	1.871	0.1149
BMI, kg/m²	23.8 ± 0.3	22.9 ± 0.3	22.6 ± 0.4	21.3 ± 0.3	23.4 ± 0.3	1.525	0.1942

Letters indicate the groups for comparison: a = compared with the HA0 group; b = compared with the HA1 group; c = compared with the HA2 group; d = compared with the HA3 group. Symbols indicate the statistical significance of differences by one-way ANOVA with Bonferroni *post hoc* correction: *P<0.05, **P<0.01, ***P<0.001, ****P<0.0001.

### 3.2 Altitude-dependent changes in CRV coupling dynamics

**Figure 1:**
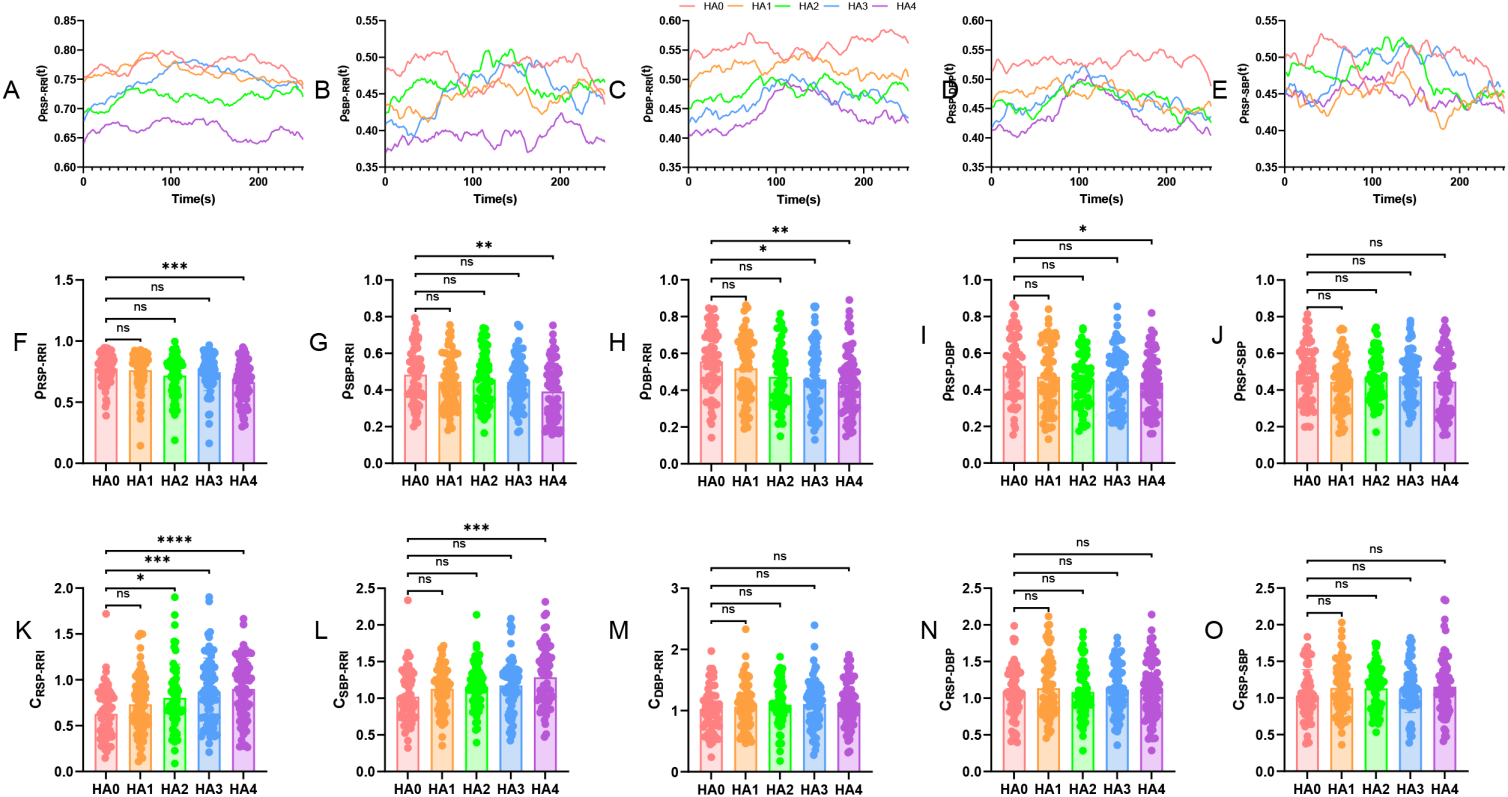
Influence of altitude gradients on CRV coupling dynamics. **(A)** dynamic characteristics of cardio-respiratory coupling ρ_RSP-RRI_(t), **(B)** dynamic characteristics of cardiac-vascular coupling ρ_SBP-RRI_(t), **(C)** dynamic characteristics of cardiac-vascular coupling ρ_DBP-RRI_(t), **(D)** dynamic characteristics of respiratory-vascular coupling ρ_RSP-DBP_(t), **(E)** dynamic characteristics of respiratory-vascular coupling ρ_RSP-SBP_(t), **(F)** Strength of cardio-respiratory coupling ρ_RSP-RRI_, **(G)** Strength of cardiac-vascular coupling ρ_SBP-RRI_, **(H)** Strength of cardiac-vascular coupling ρ_DBP-RRI_, **(I)** Strength of respiratory-vascular coupling ρ_RSP-DBP_, **(J)** Strength of respiratory-vascular coupling ρ_RSP-SBP_, **(K)** Complexity of cardio-respiratory coupling ρ_RSP-RRI_, **(L)** Complexity of cardiac-vascular coupling ρ_SBP-RRI_, **(M)** Complexity of cardiac-vascular coupling ρ_DBP-RRI_, **(N)** Complexity of respiratory-vascular coupling ρ_RSP-DBP_, **(O)** Complexity of respiratory-vascular coupling ρ_RSP-SBP_, **(P)** Total strength ${\text{ρ}}_{\text{total}}$ and complexity ${\text{C}}_{\text{total}}$ of cardio-respiratory-vascular coupling. Group differences among cohorts were evaluated by one-way ANOVA for each variable with Bonferroni *post hoc* correction: *P<0.05, **P<0.01, ***P<0.001, ****P<0.0001.

**Figure 1** illustrates the CRV coupling dynamics across the five study altitude gradients. Representative dynamic traces of the coupling parameters between respiration, heart rate, and blood pressure for each altitude are displayed in **Figures 1A–1E**. At lower altitudes (HA0, HA1), these coupling signals generally exhibited relatively stable and smooth fluctuations. As altitude increased, the amplitude and frequency of oscillations in the coupling time-series trajectories showed a tendency to intensify. Notably, at the very high altitude HA4, while the overall level of coupling parameters was reduced, their dynamic trajectories were characterized by markedly increased irregularity and unstable fluctuations.

The strength of pairwise coupling is detailed in **Figures 1F–1J**. Cardio-respiratory coupling strength ρ_RSP-RRI_ declined from 0.78 ± 0.02 in the HA0 group to 0.66 ± 0.02 in the HA4 group (P = 0.0002), representing a 15.4% reduction. The cardio-vascular coupling strength ρ_DBP-RRI_ and ρ_SBP-RRI_ followed a synchronous decreasing trend. Significant reductions in ρ_DBP-RRI_ were observed at HA3 and HA4 compared to HA0, with decreases of 17.9% (0.46 ± 0.02 *vs.* 0.56 ± 0.02, P = 0.0126) and 21.4% (0.44 ± 0.02 *vs.* 0.56 ± 0.02, P = 0.0017), respectively. Similarly, ρ_SBP-RRI_ at HA4 was 14.6% lower than at HA0 (0.39 ± 0.02 *vs.* 0.48 ± 0.02, P = 0.0024). These findings show significantly lower coupling values in the HA3 and HA4 groups compared with HA0. For respiratory-vascular coupling strength, ρ_RSP-SBP_ and ρ_RSP-DBP_ showed consistent trends, with ρ_RSP-DBP_ at HA4 demonstrating a significant 17.0% attenuation relative to HA0 (0.44 ± 0.02 *vs.* 0.53 ± 0.02, P = 0.0116). In contrast, ρ_RSP-SBP_ emerged as the most stable parameter across the entire coupling strength framework, maintaining values between 0.45 and 0.49 at all altitudes without significant inter-group differences (P>0.05).

Analysis of coupling complexity (**Figures 1K–O**) provided further insight into the differential impact of altitude on system dynamics. Cardio-respiratory coupling complexity C_RSP-RRI_ increased in a strict altitude-dependent manner, showing significant elevation at HA2 (0.80 ± 0.05 *vs.* 0.63 ± 0.04, P = 0.0396), HA3 (0.87 ± 0.04 *vs.* 0.63 ± 0.04, P = 0.0004), and HA4 (0.90 ± 0.04 *vs.* 0.63 ± 0.04, P<0.0001) compared to the HA0. For cardio-vascular coupling, complexity C_SBP-RRI_ was significantly higher only at the most very high altitude, with HA4 values exceeding those at HA0 by approximately 26% (1.29 ± 0.05 *vs.* 1.02 ± 0.05, P = 0.0001). No significant differences were observed across altitudes for the complexity metrics C_DBP-RRI_, C_RSP-DBP_, and C_RSP-SBP_ (P>0.05).

The integrated multi-system coupling metrics are summarized in **Figure 1P**. The overall coupling strength ${\text{ρ}}_{\text{total}}$ demonstrated a graded decline with increasing altitude. Starting at 1.81 ± 0.04 in HA0, it decreased to 1.70 ± 0.04 at HA1, fell further to 1.65 ± 0.04 at HA2 (P = 0.0398 *vs.* HA0), remained at 1.66 ± 0.04 at HA3 (P = 0.0402 *vs.* HA0), and reached its lowest point of 1.53 ± 0.05 at HA4 (P<0.0001 *vs.* HA0). This pattern reflects a progressive attenuation in the global linear synchrony of the CRV network. Conversely, total coupling complexity ${\text{C}}_{\text{total}}$ exhibited a clear altitude-dependent increase. Values rose from 2.69 ± 0.09 at HA0 to 2.96 ± 0.08 at HA1, reached 3.04 ± 0.09 at HA2 (P = 0.0212 *vs.* HA0), climbed to 3.13± 0.08 at HA3 (P = 0.0017 *vs.* HA0), and peaked at 3.24 ± 0.09 at HA4 (P<0.0001 *vs.* HA0).

### 3.3 HRV characteristics at different altitudes

**Figure 2:**
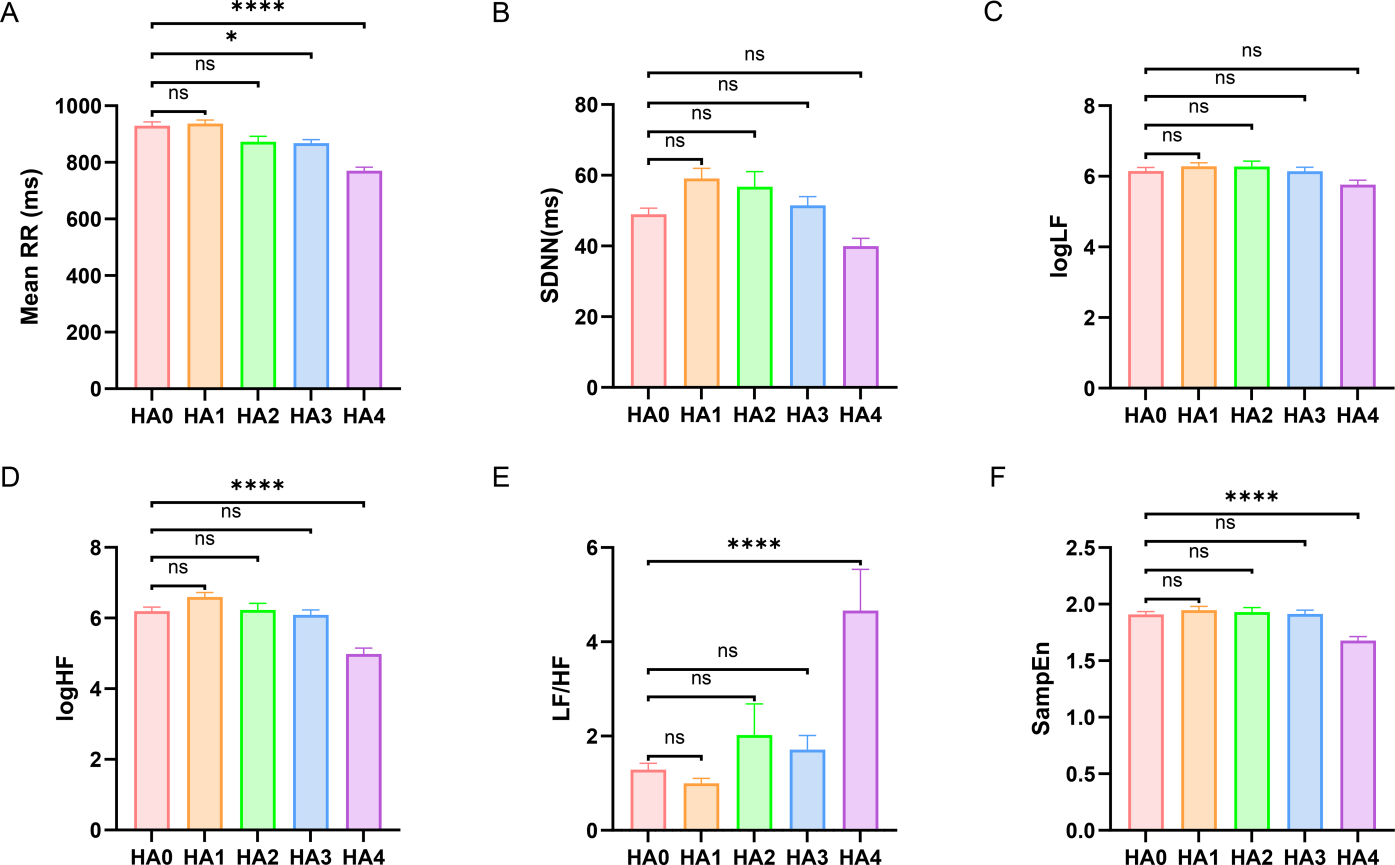
Inter-group differences in HRV indices across different altitude gradients. **(A)** Mean RR; **(B)** SDNN; **(C)** Natural logarithm of low-frequency power (logLF); **(D)** Natural logarithm of high-frequency power (logHF); **(E)** LF/HF ratio; **(F)** Sample entropy (SampEn). Group differences among cohorts were evaluated by one-way ANOVA for each variable with Bonferroni *post hoc* correction: *P<0.05, **P<0.01, ***P<0.001, ****P<0.0001.

**Figure 2** illustrates the effects of the five altitude gradients on time-domain, frequency-domain, and nonlinear measures of HRV. Mean RR progressively shortened with increasing altitude, reaching values significantly lower than those in the HA0 group at HA3 (868 ± 13 ms *vs.* 929 ± 14 ms, P = 0.0230) and HA4 (771 ± 12 ms *vs.* 929 ± 14 ms, P<0.0001). This pattern indicates a generalized heart rate acceleration under high-altitude conditions. In contrast, SDNN did not differ significantly among the altitude groups (P>0.05), suggesting that this time-domain measure was insensitive to the altitudinal gradient covered in our study and that overall time-domain HRV remained relatively stable. Regarding spectral indices, logLF showed no significant inter-group differences (P>0.05), while logHF was markedly suppressed in HA4 group compared with that of HA0 group (4.98 ± 0.17 *vs.* 6.20 ± 0.11, P<0.0001). Mirroring this change, the LF/HF ratio was substantially elevated in the HA4 group compared to HA0 (4.66 ± 0.87 *vs.* 1.29 ± 0.14, P<0.0001). As a core index of autonomic balance, the sharp rise in LF/HF indicates a shift toward sympathetic predominance at very high altitude. The nonlinear metric SampEn was also significantly reduced at HA4 compared to the lowland baseline (1.68 ± 0.04 *vs.* 1.91 ± 0.02, P<0.0001), revealing a decrease in the dynamic complexity of heart rate fluctuations.

### 3.4 Hemodynamic and respiratory changes with altitude gradients

**Figure 3:**
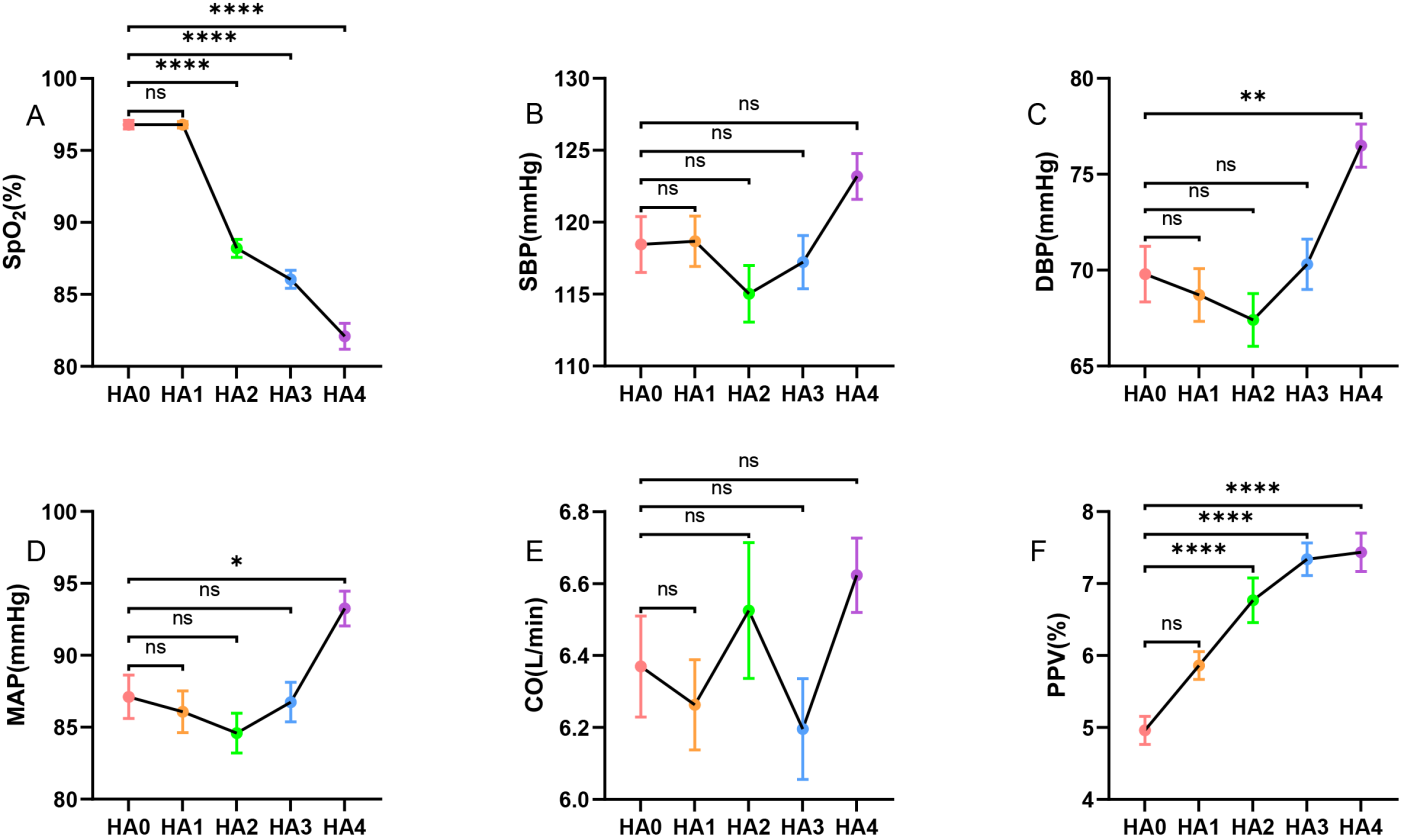
Changes in hemodynamic indices across different altitude gradients. **(A)** Oxygen saturation (SpO_2_), **(B)** systolic blood pressure (SBP), **(C)** diastolic blood pressure (DBP), **(D)** mean arterial pressure (MAP), **(E)** cardiac output (CO), and **(F)** pulse pressure variability (PPV). Group differences among cohorts were evaluated by one-way ANOVA for each variable with Bonferroni *post hoc* correction: *P<0.05, **P<0.01, ***P<0.001, ****P<0.0001.

**Figure 3** displays the altitudinal trends of key hemodynamic parameters. SpO_2_ declined in a graded manner with increasing altitude. Values were significantly lower in the HA2 (88.2 ± 0.3%, P<0.0001), HA3 (86.1 ± 0.3%, P<0.0001), and HA4 (82.1 ± 0.5%, P<0.0001) group compared to the HA0 (96.8 ± 0.2%) group. At HA4, both DBP and MAP were significantly elevated relative to HA0 levels (DBP: 76.5 ± 1.1 mmHg *vs.* 69.8 ± 1.5 mmHg, P = 0.0042; MAP: 93.3 ± 1.2 mmHg *vs.* 87.1 ± 1.5 mmHg, P = 0.0204). In contrast, SBP and CO did not show significant differences among the different altitude groups (P>0.05), suggesting that the baseline regulation of these parameters remained relatively unaltered across the studied altitude range. PPV was significantly higher in the HA2 (6.77 ± 0.31% *vs.* 4.96 ± 0.20%, P<0.0001), HA3 (7.34 ± 0.23% *vs.* 4.96 ± 0.20%, P<0.0001), and HA4 (7.44 ± 0.27% *vs.* 4.96± 0.20%, P<0.0001) groups compared to HA0.

**Figure 4:**
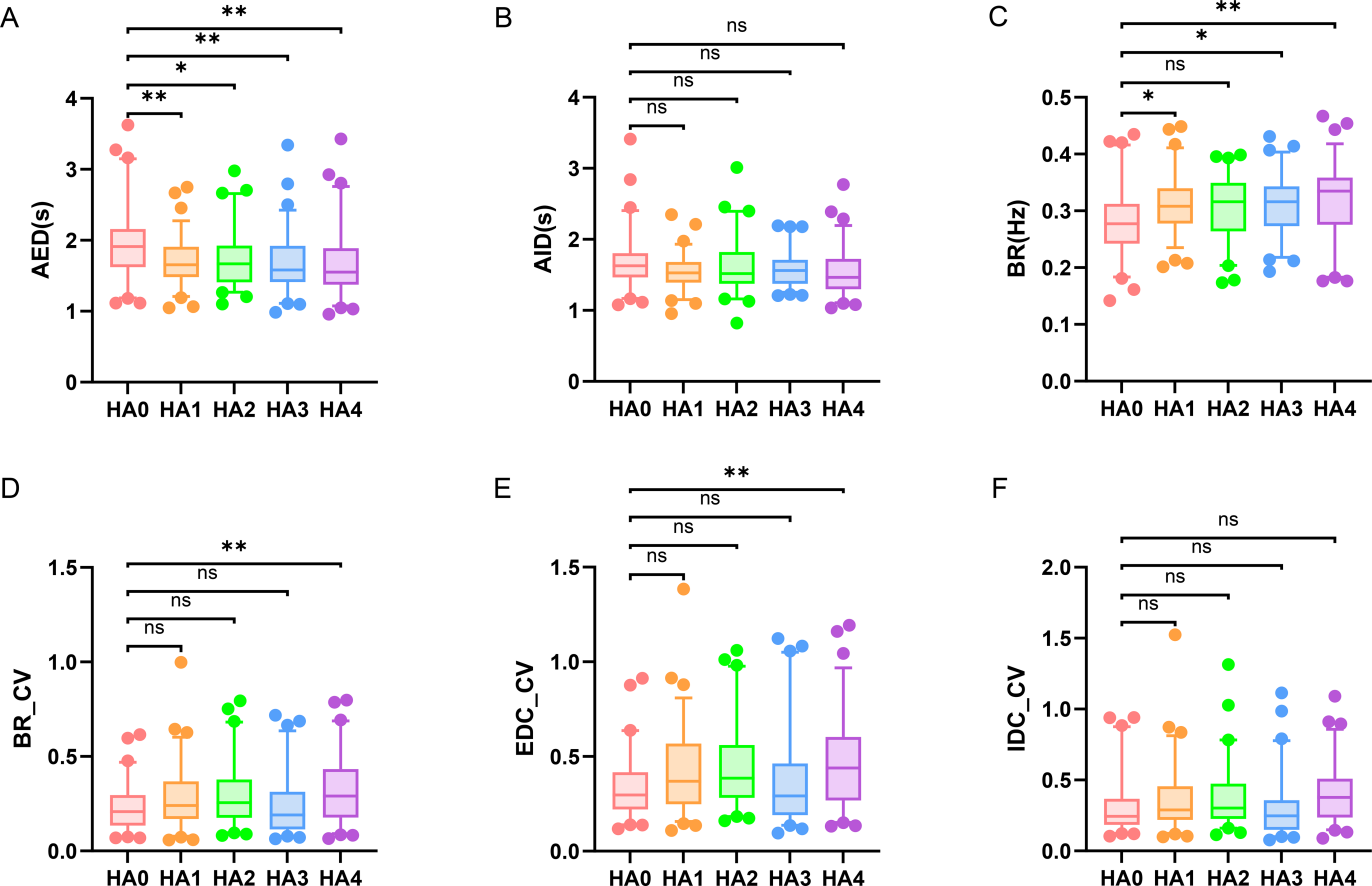
Changes in respiratory parameters across different altitude gradients. **(A)** Average exhale duration (AED), **(B)** average inhale duration (AID), **(C)** breathing rate (BR), **(D)** coefficient of variation of breathing rate (BR_CV), **(E)** coefficient of variation of exhale duty cycle (EDC_CV), and **(F)** coefficient of variation of inhale duty cycle (IDC_CV). Group differences among cohorts were evaluated by one-way ANOVA for each variable with Bonferroni *post hoc* correction: *P<0.05, **P<0.01, ***P<0.001, ****P<0.0001.

Analysis of respiratory parameters across the altitude gradient revealed a distinct, asymmetric change of breathing patterns (**Figure 4**). Regarding respiratory timing, the AED was significantly prolonged at all elevated sites compared to the lowland baseline (HA0: 1.98± 0.07 s). Specifically, AED was greater in HA1 (1.69 ± 0.04 s, P = 0.0016), HA2 (1.73 ± 0.05 s, P = 0.0180), HA3 (1.69 ± 0.05 s, P = 0.0013), and HA4 (1.69 ± 0.06 s, P = 0.0016). A concurrent elevation in BR was also observed. Values were significantly higher than those in HA0 (0.28 ± 0.01 Hz) at HA1 (0.31 ± 0.01 Hz, P = 0.0222), HA3 (0.31 ± 0.01 Hz, P = 0.0434), and HA4 (0.32 ± 0.01 Hz, P = 0.0022). Notably, significant alterations in the variability of breathing emerged only at the very high altitude. The BR_CV and EDC_CV were both substantially higher in the HA4 group (0.32 ± 0.02 for each) compared to the HA0 group (0.23 ± 0.02 for each, P = 0.0096 for both comparisons). In contrast, AID and IDC_CV remained unchanged across all altitude gradients (P>0.05).

### 3.5 Correlation analysis of CRV coupling dynamics with physiological measures at HA4

**Figure 5:**
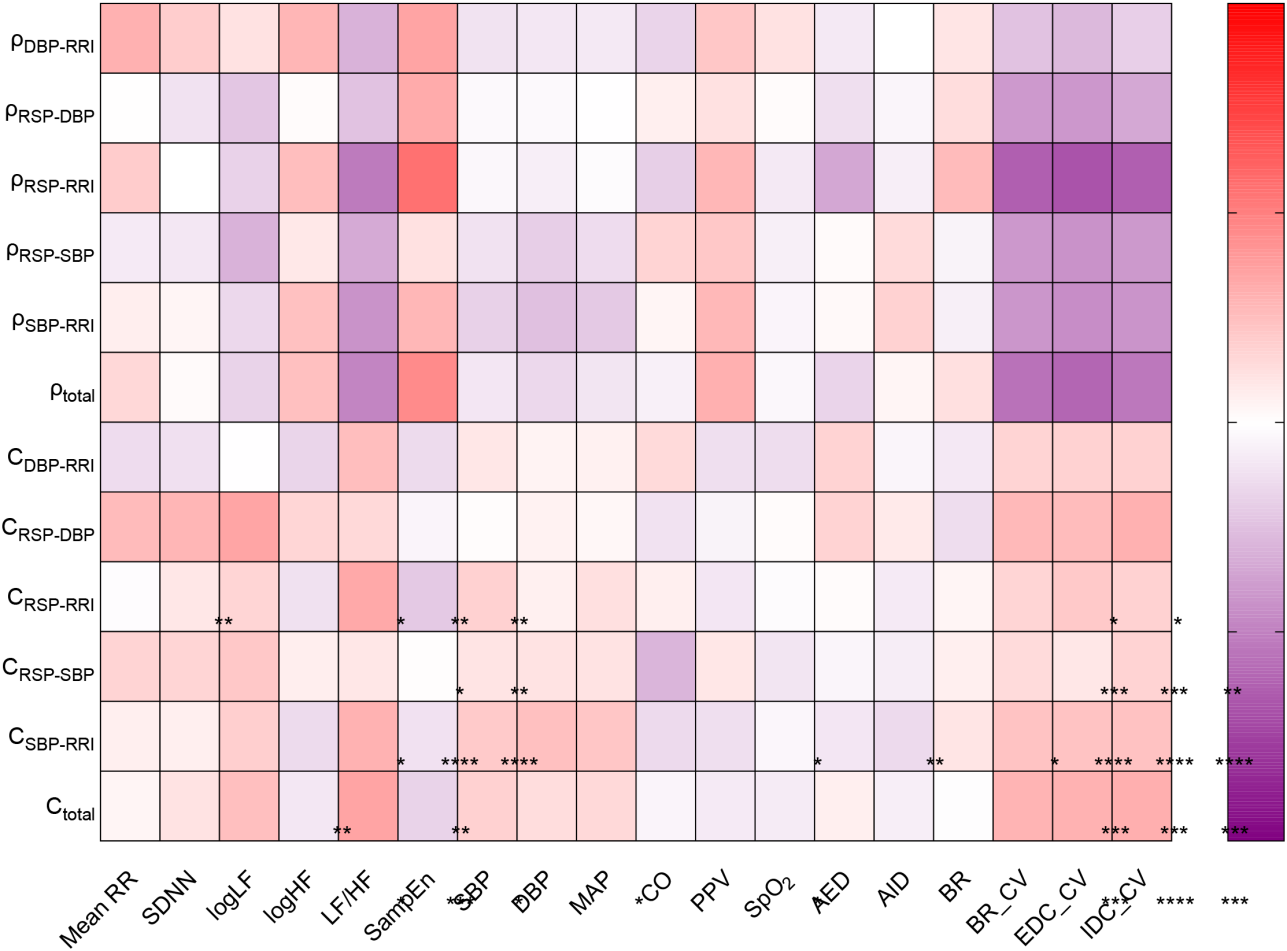
Correlation heatmap between CRV coupling indices and HRV, hemodynamics, and respiratory parameters at HA4 (5100 m). Color gradient and cell values represents Spearman correlation coefficients (ρ values). *, **, ***, and **** indicate P < 0.05, P < 0.01, P < 0.001, and P < 0.0001, respectively.

To further elucidate the integrative mechanisms linking cardiac, respiratory, and vascular systems under high-altitude stress, we performed a correlation analysis at 5100 m (HA4), examining the relationships between CRV coupling metrics and parameters derived from HRV, hemodynamics, and respiration (**Figure 5**). A consistent pattern emerged for coupling strength. Overall coupling strength ${\text{ρ}}_{\text{total}}$ and several pairwise indices (ρ_DBP-RRI_, ρ_RSP-RRI_, ρ_SBP-RRI_) showed significant positive correlations with vagally mediated logHF and with the nonlinear complexity metric SampEn. Conversely, these same coupling strength metrics were negatively correlated with the relative autonomic influence (LF/HF) and with measures of respiratory variability (BR_CV, EDC_CV). The pattern for coupling complexity was distinctly different. Overall complexity ${\text{C}}_{\text{total}}$ and specific pairwise metrics (*C*_DBP-RRI_, *C*_RSP-RRI_, *C*_SBP-RRI_) were positively correlated with LF/HF. Among all coupling metrics, cardio-respiratory coupling strength ρ_RSP-RRI_ demonstrated the broadest and strongest associative network. It was significantly correlated with multiple HRV indices (logHF: ρ=0.25, P<0.05; LF/HF: ρ=-0.52, P<0.0001; SampEn: ρ=0.55, P<0.0001), respiratory variability parameters (BR_CV: ρ=-0.63; EDC_CV: ρ=-0.68; IDC_CV: ρ=-0.63; all P<0.0001), and the hemodynamic parameter PPV (ρ=0.28, P<0.05). This extensive connectivity underscores its potential role as a central hub integrating autonomic balance, cardiac dynamics, circulatory function, and respiratory homeostasis. Notably, neither coupling strength nor coupling complexity metrics showed any significant correlation with SpO_2_ at 5100 m.

## 4 Discussion

This multi-altitude investigation systematically delineates the acclimatization-related changes of CRV coupling dynamics in populations with long-term high-altitude acclimatization. A central finding is the bimodal acclimatization characteristic wherein coupling strength attenuates in an altitude-dependent manner, while coupling complexity increases with altitude. This strength-complexity dissociation may indicate that the physiological regulation characteristic of long-term high-altitude acclimatized populations. Specifically, it may shift from reliance on precise linear synchronization toward more flexible and resilient nonlinear dynamic interactions (Bashan et al., 2012; Costa et al., 2005).

Our results demonstrate that the decline in linear coupling strength follows a distinct altitude gradient. This pattern holds for the overall coupling index ( ${\text{ρ}}_{\text{total}}$) as well as for specific pairwise measures, including cardio-respiratory (ρ_RSP-RRI_), cardio-vascular (ρ_SBP-RRI_, ρ_DBI-RRI_), and respiratory-vascular (ρ_RSP-DBP_) couplings. These observations align well with established physiological responses to high altitude, such as increased heart rate, attenuated vagal tone, and destabilized respiratory rhythm (Richalet et al., 2024; Hackett and Roach, 2001; Duplain et al., 1999; Simpson et al., 2024). Linear coupling strength reflects the instantaneous synchrony of rhythmic oscillations between systems. It is fundamental for efficient energy transfer and oxygen delivery (Nazerian et al., 2024; Qin et al., 2021; Richardson, 2003). Linear coupling strength’s attenuation may be caused by dual mechanisms. First, chronic hypoxia may impair the brain stem’s capacity to integrate rhythms of heart, lung, and vascular, leading to diminished central command synchrony (Zoccal et al., 2024; Bakker et al., 2023). Second, it may induce a blunted sensitivity of peripheral effector to neural and respiratory oscillatory drives (Michiels, 2004; Cowburn et al., 2017). Notably, the stability of ρ_RSP-SBP_ across altitudes may suggest that the mechanical modulation of systolic pressure by respiration constitutes a highly resilient pathway. This stability may be linked to its underlying neural control in that the linear coupling between respiration and systolic pressure is primarily vagally mediated (Segers et al., 2020; Rajendran et al., 2024). Despite general suppression of vagal activity during long-term acclimatization, the regulatory function dedicated to this core homeostatic pathway appears preserved. This preservation ensures a maintained circulatory response to respiratory rhythm, which may explain why systolic pressure does not change significantly across altitudes.

In contrast to the weakening linear synchrony, the complexity of system coupling exhibited a marked increase. This was evidenced by the altitude-dependent rise in overall coupling complexity ( ${\text{C}}_{\text{total}}$). The progressive elevation in cardio-respiratory coupling complexity (C_RSP-RRI_) starting from 3700 m, and the significant enhancement of cardio-vascular coupling complexity (C_SBP-RRI_) at 5100 m. Coupling complexity characterizes the diversity and unpredictability of interaction patterns between systems, with its elevation often associated with greater system resilience and adaptive regulatory capacity (Costa et al., 2005; Weng et al., 1999; Lowe, 2025). At high altitude, this increase in complexity can be interpreted as a potential compensatory mechanism. Environmental stress may compromise the system’s ability to maintain stable, precise synchrony. In response, the body broadens its regulatory repertoire by enhancing the dynamic complexity of interactions. This preserves function in a variable environment (Bashan et al., 2012). Our correlation analysis, showing positive associations between coupling complexity metrics ( ${\text{C}}_{\text{total}}$, C_RSP-RRI_, C_SBP-RRI_) and sympathetic dominance (LF/HF), supports this view. The interpretation of LF/HF as a direct measure of sympathovagal balance remains debated. Its use here is limited to a descriptive index of spectral power distribution. A moderate degree of sympathetic activation might drive higher-order systemic interactions by expanding the dynamic range and nonlinear features of central signal output (Young et al., 2021; Scott-Solomon et al., 2021).

A key regulatory insight from this study is the identification of respiratory rhythm variability (BR_CV, EDC_CV) as a strong negative modulator of coupling strength. The decline in all coupling strength indices correlated significantly with increased respiratory variability. This indicates that during high-altitude acclimatization, respiration acts not merely as a regulated variable for gas exchange but also as a fundamental stabilizer for the entire cardiovascular rhythmic network (Fisher et al., 2022; Smith et al., 2013). The rhythmic output of the respiratory center provides a crucial timing reference for cardiac and vascular oscillations (Smith et al., 2013; Ramirez et al., 2016). Long-term hypoxia-induced increases in respiratory drive and expiratory duration may disrupt the inherent rhythmic stability of the respiratory center. This instability could, in turn, propagate through the entire CRV coupling network, impairing its linear synergy (Fisher et al., 2022). Consequently, the stability of the breathing pattern itself may serve as an critical biomarker for successful high-altitude acclimation.

Furthermore, the autonomic state, reflected by the LF/HF ratio, exhibited modality-specific associations with coupling properties. Specifically, it correlated negatively with overall CRV coupling strength but positively with cardio-respiratory and cardio-vascular coupling complexity. This reveals a dual role for autonomic balance in shaping coupling dynamics (Quarti-Trevano et al., 2021; Komisaruk and Frangos, 2022). A vagally dominant state (low LF/HF) appears conducive to finely-tuned, high-synchrony linear coupling. This may be related to the vagus nerve’s capacity for direct and rapid phase-resetting of the sinoatrial node (Komisaruk and Frangos, 2022; Ma et al., 2025). Conversely, a state of relative sympathetic dominance (high LF/HF), while disruptive to linear synchrony, may enhance nonlinear inter-system interactions by increasing the complexity of central sympathetic outflow and altering vascular response dynamics (Scott-Solomon et al., 2021; Quarti-Trevano et al., 2021). This trade-off reflects the body’s dynamic adjustment between efficiency-prioritizing and resilience-prioritizing strategies in response to hypoxic stress.

An intriguing finding is the lack of significant correlation between any coupling metric and SpO_2_ at 5100 m. This challenges a simplistic linear model where the degree of hypoxia directly dictates the extent of physiological dysregulation (Saito et al., 2005; Yuri et al., 2025). It suggests a transition in the CRV coupling network following long-term acclimatization. The network may shift from directly responding to the immediate hypoxic stimulus. Instead, it may operate as a regulatory steady state established over months of remodeling, rather than a reaction to instantaneous fluctuations in oxygenation (Richalet et al., 2024; Zhang et al., 2025). This decoupling may constitute a protective mechanism, preventing core physiological rhythms from being constantly overridden by the pervasive hypoxic signal, thereby preserving a degree of functional autonomy.

This study reveals the dynamic changes of CRV system coupling under high altitude hypoxia from the perspective of network physiology, and provides new experimental evidence for understanding the coordinated regulation law of physiological network under hypoxic acclimatization (Bartsch et al., 2015). Our study has several limitations. First, the cohort consisted exclusively of healthy Han Chinese males, limiting the generalizability of findings across ethnicities. Sex-related differences in autonomic and cardiorespiratory regulation are well documented and may influence CRV coupling dynamics. The current findings cannot be generalized to females. Second, the three-month residency criterion does not necessarily reflect full physiological adaptation, which may require longer durations or lifelong exposure. Therefore, our findings are best interpreted as steady-state characteristics among individuals with established residency at each altitude, but cannot delineate the dynamic trajectory from short-term exposure to long-term acclimatization. Third, the coupling analysis is based on relationships between 5-minute duration of physiological signals. It lacks integration with molecular mechanisms, hindering a complete, multi-level understanding of the regulatory pathways underlying coupling adaptation. Although 5-minute signal length is standard for HRV, longer recording time may further improve the robustness of nonlinear complexity estimation. Fourth, by focusing on physiological parameters across altitudes, we did not correlate these with behavioral or functional performance metrics, restricting our ability to fully link multi-system change to functional output. This is a multicenter cross-sectional study, and the results only reveal the correlation between altitude gradient and CRV coupling characteristics. The causal mechanism of dynamic high-altitude acclimatization needs to be verified by longitudinal studies.

## 5 Conclusions

This cross-sectional study demonstrates altitude-associated dual-mode changes in the CRV coupling system of populations with long-term high-altitude acclimatization, characterized by attenuated linear coupling strength and potential compensatory augmentation of nonlinear system complexity. We further identify autonomic balance and the stability of respiratory rhythm as central regulatory targets governing this acclimatization process. These findings reflect cross-sectional steady-state differences rather than longitudinal adaptive trajectories. Beyond deepening the fundamental understanding of the physiological essence of high-altitude acclimatization, these insights may provide novel system-level physiological indicators and a theoretical framework for evaluating individual acclimatization capacity and implementing early warning of high-altitude decompensation risk.

## Data Availability

The raw data supporting the conclusions of this article will be made available by the authors, without undue reservation.

## References

[B1] Bakker M. E. Djerourou I. Belanger S. Lesage F. Vanni M. P. (2023). Alteration of functional connectivity despite preserved cerebral oxygenation during acute hypoxia. Sci. Rep. 13, 13269. doi: 10.1038/s41598-023-40321-3. PMID: 37582847 PMC10427674

[B2] Bartsch R. P. Liu K. K. Bashan A. Ivanov P. (2015). Network physiology: How organ systems dynamically interact. PloS One 10, e0142143. doi: 10.1371/journal.pone.0142143. PMID: 26555073 PMC4640580

[B3] Bartsch R. P. Schumann A. Y. Kantelhardt J. W. Penzel T. Ivanov P. (2012). Phase transitions in physiologic coupling. Proc. Natl. Acad. Sci. U.S.A. 109, 10181–10186. doi: 10.1073/pnas.1204568109. PMID: 22691492 PMC3387128

[B5] Bärtsch P. Swenson E. R. (2013). Clinical practice: Acute high-altitude illnesses. N. Engl. J. Med. 368, 2294–2302. doi: 10.1056/NEJMcp1214870. PMID: 23758234

[B4] Bashan A. Bartsch R. P. Kantelhardt J. W. Havlin S. Ivanov P. C. (2012). Network physiology reveals relations between network topology and physiological function. Nat. Commun. 3, 702. doi: 10.1038/ncomms1705. PMID: 22426223 PMC3518900

[B6] Burtscher M. Kleinsasser A. Burtscher J. Swenson E. R. (2025). Mechanisms of ventilatory acclimatization to high altitude. Natl. Sci. Rev. 12, nwaf244. doi: 10.1093/nsr/nwaf244. PMID: 40917648 PMC12409620

[B7] Castiglioni P. Merati G. Parati G. Faini A. (2023). Sample, fuzzy and distribution entropies of heart rate variability: What do they tell us on cardiovascular complexity. Entropy (Basel). 25, 281. doi: 10.3390/e25020281. PMID: 36832650 PMC9954876

[B8] Censi F. Calcagnini G. Strano S. Bartolini P. Barbaro V. (2003). Nonlinear coupling among heart rate, blood pressure, and respiration in patients susceptible to neuromediated syncope. Ann. Biomed. Eng. 31, 1097–1105. doi: 10.1114/1.1603748. PMID: 14582612

[B9] Cornolo J. Mollard P. Brugniaux J. V. Robach P. Richalet J. P. (2004). Autonomic control of the cardiovascular system during acclimatization to high altitude: effects of sildenafil. J. Appl. Physiol. (1985). 97, 935–940. doi: 10.1152/japplphysiol.00239.2004. PMID: 15145924

[B10] Costa M. Goldberger A. L. Peng C. K. (2005). Multiscale entropy analysis of biological signals. Phys. Rev. E. Stat. Nonlin. Soft. Matter. Phys. 71, 21906. doi: 10.1103/PhysRevE.71.021906. PMID: 15783351

[B11] Cowburn A. S. Macias D. Summers C. Chilvers E. R. Johnson R. S. (2017). Cardiovascular adaptation to hypoxia and the role of peripheral resistance. elife 6, e28755. doi: 10.7554/eLife.28755. PMID: 29049022 PMC5648530

[B12] Crystal G. J. Pagel P. S. (2020). The physiology of oxygen transport by the cardiovascular system: evolution of knowledge. J. Cardiothorac. Vasc. Anesth. 34, 1142–1151. doi: 10.1053/j.jvca.2019.12.029. PMID: 31948889

[B13] Duplain H. Vollenweider L. Delabays A. Nicod P. Bärtsch P. Scherrer U. (1999). Augmented sympathetic activation during short-term hypoxia and high-altitude exposure in subjects susceptible to high-altitude pulmonary edema. Circulation 99, 1713–1718. doi: 10.1161/01.cir.99.13.1713. PMID: 10190881

[B14] Fisher J. P. Zera T. Paton J. F. R. (2022). Respiratory-cardiovascular interactions. Handb. Clin. Neurol. 188, 279–308. doi: 10.1016/B978-0-323-91534-2.00006-0. PMID: 35965029

[B15] Furian M. Bitos K. Hartmann S. E. Muralt L. Lichtblau M. Bader P. R. . (2022). Acute high altitude exposure, acclimatization and re-exposure on nocturnal breathing. Front. Physiol. 13. doi: 10.3389/fphys.2022.965021. PMID: 36134332 PMC9483161

[B16] Garcia-Retortillo S. Gacto M. O’Leary T. J. Noon M. Hristovski R. Balagué N. . (2019). Cardiorespiratory coordination reveals training-specific physiological adaptations. Eur. J. Appl. Physiol. 119, 1701–1709. doi: 10.1007/s00421-019-04160-3. PMID: 31187282

[B17] Gatterer H. Villafuerte F. C. Ulrich S. Bhandari S. S. Keyes L. E. Burtscher M. (2024). Altitude illnesses. Nat. Rev. Dis. Primers 10, 43. doi: 10.1038/s41572-024-00526-w. PMID: 38902312

[B18] Hackett P. H. Roach R. C. (2001). High-altitude illness. N. Engl. J. Med. 345, 107–114. doi: 10.1056/NEJM200107123450206. PMID: 11450659

[B19] Hermann B. (2024). Aberrant brain-heart coupling is associated with the severity of post cardiac arrest brain injury. Ann. Clin. Transl. Neurol. 11, 866–882. doi: 10.1002/acn3.52000. PMID: 38243640 PMC11021613

[B20] Hou J. Lu K. Chen P. Wang P. Li J. Yang J. . (2023). Comprehensive viewpoints on heart rate variability at high altitude. Clin. Exp. Hypertens. 45, 2238923. doi: 10.1080/10641963.2023.2238923. PMID: 37552638

[B21] Ivanov P. C. (2021). The new field of network physiology: Building the human physiolome. Front. Netw. Physiol. 1. doi: 10.3389/fnetp.2021.711778. PMID: 36925582 PMC10013018

[B22] Kaminsky D. A. Cockcroft D. W. Davis B. E. (2023). Respiratory system dynamics. Semin. Respir. Crit. Care Med. 44, 526–537. doi: 10.1055/s-0043-1770058. PMID: 37429331

[B23] Kitney R. I. Fulton T. McDonald A. H. Linkens D. A. (1985). Transient interactions between blood pressure, respiration and heart rate in man. J. Biomed. Eng. 7, 217–224. doi: 10.1016/0141-5425(85)90022-6. PMID: 4033097

[B24] Komisaruk B. R. Frangos E. (2022). Vagus nerve afferent stimulation: Projection into the brain, reflexive physiological, perceptual, and behavioral responses, and clinical relevance. Auton. Neurosci. 237, 102908. doi: 10.1016/j.autneu.2021.102908. PMID: 34823149

[B25] Leacy J. K. Linares A. M. Zouboules S. M. Rampuri Z. H. Bird J. D. Herrington B. A. . (2021). Cardiorespiratory hysteresis during incremental high-altitude ascent-descent quantifies the magnitude of ventilatory acclimatization. Exp. Physiol. 106, 139–150. doi: 10.1113/EP088488. PMID: 32421248

[B26] Li B. N. Dong M. C. Vai M. I. (2010). On an automatic delineator for arterial blood pressure waveforms. Biomed. Signal. Proces. 5, 76–81. doi: 10.1016/j.bspc.2009.06.002. PMID: 41936479

[B27] Lin C. Lin P. F. Wang C. H. Juan C. H. Tran T. T. Pham V. T. . (2020). Probing age-related changes in cardio-respiratory dynamics by multimodal coupling assessment. Chaos 30, 033118. doi: 10.1063/1.5134868. PMID: 32237792

[B28] Liu H. Zhan P. Shi J. Hu M. Wang G. Wang W. (2020). Heart rhythm complexity as predictors for the prognosis of end-stage renal disease patients undergoing hemodialysis. BMC. Nephrol. 21, 536. doi: 10.1186/s12882-020-02196-8. PMID: 33297978 PMC7727237

[B29] Lowe S. R. (2025). Embracing complexity in resilience research. Nat. Mental. Health 3, 391–392. doi: 10.1038/s44220-025-00403-9. PMID: 41933181

[B30] Luks A. M. Swenson E. R. Bärtsch P. (2017). Acute high-altitude sickness. Eur. Respir. Rev. 26, 160096. doi: 10.1183/16000617.0096-2016. PMID: 28143879 PMC9488514

[B5001] Luks A. M. Beidleman B. A. Freer L. Grissom C. K. Keyes L. E. McIntosh S. E. . (2024). Wilderness Medical Society Clinical Practice Guidelines for the Prevention, Diagnosis, and Treatment of Acute Altitude Illness: 2024 Update. Wilderness. Environ. Med. 35, 2S–19S. doi: 10.1016/j.wem.2023.05.013, PMID: 37833187

[B31] Ma D. S. Li L. Shi W. B. Li M. W. Zhang J. Fan Y. . (2024). Multimodal coupling and HRV assessment characterize autonomic functional changes in congestive heart failure patients with sinus rhythm or severe arrhythmia. Biomed. Signal. Proces. 89, 105764. doi: 10.1016/j.bspc.2023.105764. PMID: 41936479

[B32] Ma L. Wang H. B. Hashimoto K. (2025). The vagus nerve: An old but new player in brain-body communication. Brain. Behav. Immun. 124, 28–39. doi: 10.1016/j.bbi.2024.11.023. PMID: 39566667

[B33] Menuet C. Ben-Tal A. Linossier A. Allen A. M. MaChado B. H. Moraes D. J. A. . (2025). Redefining respiratory sinus arrhythmia as respiratory heart rate variability: an international Expert Recommendation for terminological clarity. Nat. Rev. Cardiol. 22, 978–984. doi: 10.1038/s41569-025-01160-z. PMID: 40328963

[B34] Michiels C. (2004). Physiological and pathological responses to hypoxia. Am. J. Pathol. 164, 1875–1882. doi: 10.1016/S0002-9440(10)63747-9. PMID: 15161623 PMC1615763

[B35] Mohanty S. Ahmad Y. (2025). Recent updates on sickness during acute high-altitude hypoxic exposure and its management. Adv. Redox Res. 15, 100127. doi: 10.1016/j.arres.2025.100127. PMID: 41936479

[B36] Narvaez-Guerra O. Herrera-Enriquez K. Medina-Lezama J. Chirinos J. A. (2018). Systemic hypertension at high altitude. Hypertension 72, 567–578. doi: 10.1161/HYPERTENSIONAHA.118.11140. PMID: 30354760 PMC6205208

[B37] Nazerian A. Hart J. D. Lodi M. Sorrentino F. (2024). The efficiency of synchronization dynamics and the role of network syncreactivity. Nat. Commun. 15, 9003. doi: 10.1038/s41467-024-52486-0. PMID: 39424789 PMC11489704

[B5000] Nuss R . (2022). Medical conditions and high-altitude travel. N. Engl. J. Med. 386, 364–373. doi: 10.1056/NEJMc2203182, PMID: 35544404

[B38] Ortiz-Prado E. Kumar Sethy N. Vasconez-Gonzalez J. Izquierdo-Condoy J. S. (2024). Editorial: Integrative physiological approaches to understand high altitude adaptation. Front. Physiol. 15. doi: 10.3389/fphys.2024.1487290. PMID: 39308976 PMC11412832

[B39] Parati G. Agostoni P. Basnyat B. Bilo G. Brugger H. Coca A. . (2018). Clinical recommendations for high altitude exposure of individuals with pre-existing cardiovascular conditions: A joint statement by the European Society of Cardiology, the Council on Hypertension of the European Society of Cardiology, the European Society of Hypertension, the International Society of Mountain Medicine, the Italian Society of Hypertension and the Italian Society of Mountain Medicine. Eur. Heart. J. 39, 1546–1554. doi: 10.1093/eurheartj/ehx720. PMID: 29340578 PMC5930248

[B40] Prabhakar N. R. Semenza G. L. (2015). Oxygen sensing and homeostasis. Physiol. (Bethesda). 30, 340–348. doi: 10.1152/physiol.00022.2015. PMID: 26328879 PMC4556828

[B41] Prosperi P. Verratti V. Taverna A. Rua R. Bonan S. Rapacchiale G. . (2023). Ventilatory function and oxygen delivery at high altitude in the Himalayas. Respir. Physiol. Neurobiol. 314, 104086. doi: 10.1016/j.resp.2023.104086. PMID: 37257573

[B42] Qin B. W. Zhao L. Lin W. (2021). A frequency-amplitude coordinator and its optimal energy consumption for biological oscillators. Nat. Commun. 12, 5894. doi: 10.1038/s41467-021-26182-2. PMID: 34625549 PMC8501100

[B43] Quarti-Trevano F. Seravalle G. Grassi G. (2021). Clinical relevance of the sympathetic-vascular interactions in health and disease. Biomedicines 9, 1007. doi: 10.3390/biomedicines9081007. PMID: 34440211 PMC8394495

[B44] Rajendran P. S. Hadaya J. Khalsa S. S. Yu C. Chang R. Shivkumar K. (2024). The vagus nerve in cardiovascular physiology and pathophysiology: From evolutionary insights to clinical medicine. Semin. Cell Dev. Biol. 156, 190–200. doi: 10.1016/j.semcdb.2023.01.001. PMID: 36641366 PMC10336178

[B45] Ramirez J. M. Dashevskiy T. Marlin I. A. Baertsch N. (2016). Microcircuits in respiratory rhythm generation: commonalities with other rhythm generating networks and evolutionary perspectives. Curr. Opin. Neurobiol. 41, 53–61. doi: 10.1016/j.conb.2016.08.003. PMID: 27589601 PMC5495096

[B47] Richalet J. P. Hermand E. Lhuissier F. J. (2024). Cardiovascular physiology and pathophysiology at high altitude. Nat. Rev. Cardiol. 21, 75–88. doi: 10.1038/s41569-023-00924-9. PMID: 37783743

[B46] Richardson R. S. (2003). Oxygen transport and utilization: an integration of the muscle systems. Adv. Physiol. Educ. 27, 183–191. doi: 10.1152/advan.00038.2003. PMID: 14627616

[B48] Romano C. Innocenti L. Schena E. Sacchetti M. Nicolò A. Massaroni C. (2023). A signal quality index for improving the estimation of breath-by-breath respiratory rate during sport and exercise. IEEE Sens. J. 23, 31250–31258. doi: 10.1109/JSEN.2023.3330444. PMID: 41116384

[B49] Saito S. Tanobe K. Yamada M. Nishihara F. (2005). Relationship between arterial oxygen saturation and heart rate variability at high altitudes. Am. J. Emerg. Med. 23, 8–12. doi: 10.1016/j.ajem.2004.09.023. PMID: 15672330

[B50] Scott-Solomon E. Boehm E. Kuruvilla R. (2021). The sympathetic nervous system in development and disease. Nat. Rev. Neurosci. 22, 685–702. doi: 10.1038/s41583-021-00523-y. PMID: 34599308 PMC8530968

[B51] Segers L. S. Nuding S. C. Ott M. M. O’Connor R. Morris K. F. Lindsey B. G. (2020). Blood pressure drives multispectral tuning of inspiration via a linked-loop neural network. J. Neurophysiol. 124, 1676–1697. doi: 10.1152/jn.00442.2020. PMID: 32965158 PMC7814902

[B52] Simpson L. L. Stembridge M. Siebenmann C. Moore J. P. Lawley J. S. (2024). Mechanisms underpinning sympathoexcitation in hypoxia. J. Physiol. 602, 5485–5503. doi: 10.1113/JP284579. PMID: 38533641

[B53] Smith J. C. Abdala A. P. Borgmann A. Rybak I. A. Paton J. F. (2013). Brainstem respiratory networks: building blocks and microcircuits. Trends Neurosci. 36, 152–162. doi: 10.1016/j.tins.2012.11.004. PMID: 23254296 PMC4080795

[B55] Storz J. F. (2021). High-altitude adaptation: Mechanistic insights from integrated genomics and physiology. Mol. Biol. Evol. 38, 2677–2691. doi: 10.1093/molbev/msab064. PMID: 33751123 PMC8233491

[B54] Storz J. F. Scott G. R. (2019). Life ascending: Mechanism and process in physiological adaptation to high-altitude hypoxia. Annu. Rev. Ecol. Evol. Syst. 50, 503–526. doi: 10.1146/annurev-ecolsys-110218-025014. PMID: 33033467 PMC7540626

[B56] Suth D. Luther S. Lilienkamp T. (2024). Chaos control in cardiac dynamics: terminating chaotic states with local minima pacing. Front. Netw. Physiol. 4. doi: 10.3389/fnetp.2024.1401661. PMID: 39022296 PMC11252590

[B57] Task Force of the European Society of Cardiology the North American Society of Pacing (1996). Heart rate variability: standards of measurement, physiological interpretation, and clinical use. Circulation 93, 1043–1065. 8598068

[B58] Tikhonova I. V. Grinevich A. A. Tankanag A. V. (2022). Analysis of phase interactions between heart rate variability, respiration and peripheral microhemodynamics oscillations of upper and lower extremities in human. Biomed. Signal. Proces. 71, 103091. doi: 10.1016/j.bspc.2021.103091. PMID: 41936479

[B59] Tse G. Wong S. T. Tse V. Lee Y. T. Lin H. Y. Yeo J. M. (2016). Cardiac dynamics: Alternans and arrhythmogenesis. J. Arrhythm. 32, 411–417. doi: 10.1016/j.joa.2016.02.009. PMID: 27761166 PMC5063258

[B60] Wang C. Zhang L. Liu Z. Chen Z. Li Y. Fu Y. . (2025). Effects of long-term very high-altitude exposure on cardiopulmonary function of healthy adults in plain areas. Sci. Rep. 15, 24826. doi: 10.1038/s41598-025-07474-9. PMID: 40640248 PMC12246193

[B61] Wang Y. Zhang Q. Ma Q. Wang Q. Huang D. Ji X. (2024). Intermittent hypoxia preconditioning can attenuate acute hypoxic injury after a sustained normobaric hypoxic exposure: A randomized clinical trial. CNS Neurosci. Ther. 30, e14662. doi: 10.1111/cns.14662. PMID: 38477221 PMC10934266

[B62] Weng G. Bhalla U. S. Iyengar R. (1999). Complexity in biological signaling systems. Science 284, 92–96. doi: 10.1126/science.284.5411.92. PMID: 10102825 PMC3773983

[B63] Young B. E. Greaney J. L. Keller D. M. Fadel P. J. (2021). Sympathetic transduction in humans: recent advances and methodological considerations. Am. J. Physiol. Heart Circ. Physiol. 320, H942–H953. doi: 10.1152/ajpheart.00926.2020. PMID: 33416453 PMC7988755

[B64] Yuri E. Chung H. Y. Chen F. S. (2025). Reframing SpO2 tolerance as a physiological switch: implications for hypoxic adaptation and exercise regulation. Front. Physiol. 16. doi: 10.3389/fphys.2025.1667238. PMID: 40969915 PMC12441037

[B65] Zhang X. Zhang Z. Ye R. Meng Q. Chen X. (2022). Prevalence of hypertension and its relationship with altitude in highland areas: a systematic review and meta-analysis. Hypertens. Res. 45, 1225–1239. doi: 10.1038/s41440-022-00955-8. PMID: 35705740

[B5002] Zhang X. Yang Y. Shi Q . (2025). DNA methylation in adaptation to high-altitude environments and pathogenesis of related diseases. Hum. genomics. 19, 100. doi: 10.1186/s40246-025-00794-x, PMID: 40886024 PMC12398096

[B66] Zoccal D. B. Vieira B. N. Mendes L. R. Evangelista A. B. Leirão I. P. (2024). Hypoxia sensing in the body: An update on the peripheral and central mechanisms. Exp. Physiol. 109, 461–469. doi: 10.1113/EP091206. PMID: 38031809 PMC10988761

